# Supporting children’s mental health in primary schools: a qualitative exploration of educator perspectives

**DOI:** 10.1007/s13384-022-00558-9

**Published:** 2022-08-18

**Authors:** Alison Giles-Kaye, Jon Quach, Frank Oberklaid, Meredith O’Connor, Simone Darling, Georgia Dawson, Ann-Siobhan Connolly

**Affiliations:** 1 Centre for Community Child Health, Murdoch Children’s Research Institute, Children’s Hospital, RoyalMelbourne, VIC Australia; 2 The Royal Children’s Hospital, Melbourne, VIC Australia; 3 Melbourne Graduate School of Education, University of Melbourne, Melbourne, VIC, Australia; 4 Department of Pediatrics, University of Melbourne, VIC, Australia

**Keywords:** Child mental health, School, Educators, Primary, Qualitative

## Abstract

Schools are often seen as a key setting for the provision of mental health support for children. This study aimed to explore the professional perspectives of primary school educators in Victoria, Australia, regarding how schools can support the mental health of their students. Semi-structured interviews and focus groups were conducted with 17 primary school educators, from four schools. Thematic analysis was used to generate themes from the data. This study indicates that educators report significant concerns about the complexities of their role and their capacity to support children’s mental health due to a lack of resources, overwhelming demands, and inadequate training. Educators highlighted the importance of partnership and communication with families and of a school culture that prioritises mental health. This study provides insights into external factors that can undermine effective support of children’s mental health within primary schools and indicates a need for a more integrated approach to supporting children’s mental health across education and healthcare.

## Introduction

Good mental health is defined by the World Health Organization as “a state of wellbeing in which every individual realises his or her own potential, can cope with the normal stresses of life, can work productively and fruitfully and is able to make a contribution to her or his community” (World Health Organization & Calouste Gulbenkian Foundation, 2014). This understanding of mental health speaks to the presence of functional skills and strategies as well as emotional states. According to a large national survey of parents and adolescents, over half a million (13.6%) Australian 4- to 11-year-olds experience a diagnosable mental health disorder each year, and many more children are estimated to experience subclinical mental health difficulties (Johnson et al., 2018; Lawrence et al., 2015). Left unaddressed, the early life experience of mental illness is associated with later recurrence, compromised social relationships, unemployment and general ill-health (Danby & Hamilton, 2016; Goldstein & Morewitz, 2011; Green et al., 2013).

The recent Australian Productivity Commission (2020) and the Royal Commission into Victoria’s Mental Health System (2021) have revealed a system that is crisis driven and overwhelmed, where appropriate support is too often inaccessible (Productivity Commission, 2020; State of Victoria, 2021). This is particularly the case for children’s mental health. Both commissions advocate shifting to a focus on mental health promotion and prevention and focussing on schools as a setting for identifying and supporting children’s mental health needs (Productivity Commission, 2020; State of Victoria, 2021). It is well established that early intervention is more effective than treatment once symptoms become entrenched and that the first few years of primary school are a key period when risk and protective factors can profoundly impact the trajectory of children’s mental health (Cefai & Camilleri, 2015; Centre for Community Child Health, 2006, 2012; Marryat et al., 2018). The knowledge that half of adult mental health disorders emerge before 14 years of age (Birleson & Vance, 2008; Fernando et al., 2018), further speaks to the importance of addressing children’s mental health needs during their early primary school years so that they can experience optimal learning and wellbeing throughout their lives.

Motivation to address children’s mental health difficulties early, coupled with the fact that nearly all children attend a primary school (Lawrence et al., 2015), has meant that primary schools have increasingly been seen as a key community setting in which to support children’s mental health (Burns & Rapee, 2019; Fabiano & Evans, 2019; Patalay et al., 2014, 2017; Werner-Seidler et al., 2017). However, a theme throughout previous research is the lack of training and support teachers in general receive and the low confidence they feel about supporting children’s mental health (Anderson et al., 2019; Andrews et al., 2014; Yamaguchi et al., 2020). Teachers have concerns that the mental health needs of children in primary schools far outweigh their professional capabilities and the resources available to support them (Australian Education Union, 2019; Graham et al., 2011; Hussein & Vostanis, 2013; Kratt, 2017; Yamaguchi et al., 2020). While previous literature conceptualises schools as an ideal venue to support children’s mental health, there is limited understanding of what that might look like within schools and for educators. If primary schools are to be charged with the responsibility of supporting children’s mental health, then, to inform and sustain policy direction, it is important to ascertain primary school educators’ views in this regard.

In Victoria, 41% of primary school teachers have reported that they have students with mental health difficulties in their class (Australian Education Union, 2019). These children experience a range of symptoms that inhibit their daily functioning and wellbeing (Fabiano & Evans, 2019; Forns et al., 2011; Tandon et al., 2009). In a school setting, these difficulties impact not only the individual child but also their peers and school staff, inhibiting learning and teaching.

Some research in the United Kingdom and Australia has been undertaken to explore educators’ perspectives. However, these have mainly focussed on secondary schools (Graham et al., 2011; Kidger et al., 2010; Mazzer & Rickwood, 2015; Shelemy et al., 2019). Generally, these data indicated that educators thought it was important to support student mental health but felt constrained because they lacked effective training, experienced low confidence, and were frustrated by inaccessible child mental health services. While the perspectives of secondary school teachers may well resonate with primary school educators, there is a lack of qualitative empirical data to confirm this. Primary school educators may have different challenges, especially as the students are younger and may have different functional characteristics to indicate differences in mental wellbeing as well as different types of developmentally appropriate responses and supports available. The role of a secondary school teacher, who teaches subject-specific cohorts, differs substantially from the primary school teacher, who teaches year level cohorts across all subjects and therefore spends substantially more time with their students, likely gaining a different perspective. Results from survey data provide quantitative insights into educator perspectives; however, these data to date lack the rich insights that can be gained from qualitative investigation. Quantitative data are restrained by the questions asked by the survey, with forced options and little opportunity to expand on responses, whereas qualitative methods allow for a more exploratory approach to the topic where participants can initiate discussion on issues. Further, it is important to explore primary school educators’ views in order to sustain policy and practice in primary schools regarding universal and targeted prevention and intervention strategies. For these reasons, it is highly complementary to have the addition of qualitative data. This study was designed to elicit qualitative perspectives from educators in primary schools in Australia to understand their views on how they think schools can best support children’s mental health.

## Methods

This study aimed to generate insights into the perspectives of primary school educators about how schools can support children’s mental health, focussing on Foundation to Grade 3. This was a qualitative inquiry using interviews and focus groups to explore the professional experiences and perspectives of Victorian state primary school educators, experienced in teaching Foundation to Grade 3. Thematic analysis was employed to generate themes and subthemes. Ethical approval for the study was granted by the Royal Children’s Human Research Ethics Committee (HREC 57,564) and the Department of Education and Training, Victoria (2019_004161). The study was guided by the Consolidated Criteria for Reporting Qualitative research (COREQ): A 32-item checklist for interviews and focus groups to support rigour and transparency (Tong et al., 2007)(Appendix 1).

## Participants

We used a purposive sampling technique to recruit educators in mainstream Victorian state primary schools, and convenience sampling for pragmatic reasons of geographic location, time and accessibility (Kervin et al., 2006). Primary school leaders and classroom teachers were eligible to participate in the study if they had current or recent experience in Foundation to Grade 3. Recruitment was focussed in one region of the Victorian Department of Education and Training (DET). The study was advertised by DET to all primary schools in two areas of the region. Eligible participants contacted the researcher to express their interest and were provided with further written information about the study.

Four schools participated in data collection between November 2019 and March 2020. In total, 17 educators participated in the study—six school leaders and 11 classroom teachers. Nine classroom teachers were working within Foundation to Year 3 and two within Year 4–6. Participants included two men and 15 women; teaching experience ranged from four to 30 years. Six in-person interviews with school leaders and four focus groups with classroom teachers were conducted. The sample size enabled the collection and analysis of in-depth data to generate themes and subthemes. One school was located in the inner city, two were suburban metropolitan and one was semi-rural.

## Procedure

Data collection was via semi-structured interviews and focus groups which were informed by a question guide (Appendix 2), written by the lead author in consultation with the research team. The question guide was piloted with several educators and researchers before it was finalised and comprised open ended questions designed to engage participants, moving from general to specific questions (Krueger & Casey, 2015). The questioning was designed to elicit participants’ general views on children’s mental health, and support of children’s mental health needs, along with barriers and enablers to support within schools. The lead author and participant interviewer is a female PhD candidate at the University of Melbourne, who is a qualified social worker and primary school teacher. The broader research team involved in the study was multi-disciplinary and included educators, psychologists, a paediatrician and an experienced qualitative researcher. To mitigate any power imbalances between leaders and teachers, school leaders were offered individual interviews, and teachers were given the option of an interview or a small focus group. The authors had no prior relationship with any of the participants. Participants provided their informed written consent before any data collection began, and data collection was conducted in a private room in the educator’s school by the lead author, with no non-participants present. Interviews and focus groups lasted between 20 and 79 min and were audio-recorded then transcribed verbatim by a transcription company.

## Data analysis

This article aimed to explore educator perspectives of how schools can support children’s mental health, and therefore, this analysis focussed on data related only to that topic. Thematic analysis was conducted inductively commencing after initial data collection (Pope et al., 2000). The lead author read and re-read each transcript to de-identify the data, check for accuracy and ensure familiarity. De-identified transcripts were imported into NVivo12 (QSR International, 2021) to support coding. The coding process was iterative with codes, categories and themes being generated through data immersion, reflection and writing (Braun & Clarke, 2019, 2021) with transcripts being coded and recoded as new codes were generated from ongoing data collection. Participant perspectives were checked by the interviewer throughout the data collection via verbal clarification techniques; however, returning transcripts for member checking was not undertaken after data collection or analysis to avoid excessive demands on participant time. To support rigour, two researchers were involved in the coding process and six in overall thematic analysis. Two researchers (AGK, ASC) independently cross-checked the codes, categories and themes. Categories and themes were discussed by the research team until the authors thought the data were effectively represented.

## Results

Four themes, each with subthemes were generated from the data: complexities of the educator role; partnership with families; school culture that prioritises mental health; and external factors that influence school capacity (Table 1).

**Table 1:** Themes and Subthemes generated from the data

Themes	Subthemes
Complexities of the educator role	Motivation to prioritise students’ social-emotional learning Feeling pressured and overwhelmed Informal strategies for identification of mental health difficulties Being flexible and responsive Clear and regular communication
Importance of partnership with families	Complexities in the communication and engagement with families Family factors
A school culture that prioritises mental health	Importance of whole-school approach Developing relationships with children Provision of school-based resources Impact on staff wellbeing
External factors that influence school capacity	Inaccessibility of support services Insufficient support staff in schools The need to improve child mental health literacy

## Complexities of the educator role

A theme from all participants was concern around the complexities of the educator’s role regarding the support of children’s mental health. There was repeated discussion about the uneasiness that educators felt that while they were trained to teach, they were increasingly being required to engage in supporting children’s mental health.

### Motivation to prioritise students’ social-emotional learning

All educators described high levels of motivation to support their students’ mental health, citing the significant impact mental health difficulties have on student learning in the classroom. Educators described the disruption to learning, for all students, when a child in the class is experiencing mental health difficulties and therefore the need to prioritise explicit teaching of social-emotional wellbeing skills and strategies to all students.

I think that’s the biggest thing, that’s the most important thing. If they’re feeling happy and they’re engaged and they’re going to learn better. [classroom teacher]

... I guess, also students with poor mental health would also—can also stop other children from learning. So, it’s pretty critical that we get that right I think. [classroom teacher]

### Feeling pressured and overwhelmed

Most educators consistently reported feeling significant pressure regarding their dual role. They expressed concern about the difficulties of balancing their teaching responsibilities with their need to support students’ mental health. Many felt that they would benefit from mental health training but said that time pressures mean they were not able to do or implement training and that students need individual mental health support that educators are not able to provide.

...we do need training, but we also don’t have time to do it...we can’t be trained in everything and be able to do everything. ...we also need someone to just come in and intervene for us sometimes. [classroom teacher]

We need more help. We’re not psychologists. So, when the kids come and say ... ‘I will kill myself,’ and we don’t know how to handle that. We don’t know what to do... [school leader]

### Informal strategies for identification of mental health difficulties

All participants reported that they tended to rely on informal strategies such as relationship building, intuition and observation of behaviour to identify and support the mental health needs of their students. Getting to know their students was seen as vital so that educators could identify when children were struggling. However, educators spoke about lacking confidence and commonly thought that effectively identifying children’s mental health needs is a complex task for which they were not trained.

...when you build a relationship with the students from your class you can kind of see when they’re having an off day—you can just tell when they’re not quite feeling right that day. [classroom teacher]

### Being flexible and responsive

A common view among several participants was the need to be flexible and responsive to student needs, particularly regarding lesson plans and schedules. Educators spoke about the need to change plans in response to student needs, often adjusting teaching plans at very short notice if they judge that students are not coping.

...sometimes you would’ve read your class and gone, you know what, I was going to do maths today, we can’t do maths today because you just are not going to be able to cope with that ... [classroom teacher]

### Clear and regular communication

Participants typically reported that clear and regular communication with stakeholders was key in identifying and supporting the mental health of their students. Systems in schools to record and promote communication about children’s needs between colleagues and from year to year were seen as vital to enable a consistent approach to support individual students.

During our staff meetings, we usually have a section of that dedicated to wellbeing of the students, so that’s the time that we bring up any students that we’re having any concerns about and then we’ll discuss them. [classroom teacher]

## Importance of partnership with families

A view common to all participants was the importance of partnering with families to support children’s mental health and the complexities and challenges involved in communication and family engagement. Participants advocated the need to develop partnerships with families to facilitate open communication and consistency in supporting students, as well as to expedite the provision of external services if needed.

### Complexities in communication and engagement with families

Educators typically spoke about the complexities of communicating and engaging with families and the need for increased literacy around children’s mental health, to support school–home partnerships and to facilitate children accessing support. Participants expressed concern about the challenges they face when families have limited understanding of children’s mental health and that this hampers their endeavours to support their students.

People come from so many different places and not everyone’s got the same culture, upbringing, values or even knowledge of what to do... Seminars and workshops for students with their parents or just the parents would be helpful. [classroom teacher]

### Family factors

Participants generally described the economic, work, health, social and emotional pressures families experience as having an influence on effective partnerships between the school and home. Educators who have been practising for many years felt that pressures on families have become more intense over that time.

...the pressures of life I think [there] seems to be much more pressure on families these days than it was back then. [school leader]

## A school culture that prioritises mental health

A common view among participants was that a school culture that prioritises mental health is vital to enable educators’ support of children’s mental health in classrooms. Educators highlighted a range of issues they saw as important, including a whole-school approach, schools valuing staff relationships with children, the provision of a range of school-based resources and prioritising staff wellbeing.

### Importance of a whole-school approach

All participants spoke about the necessity of a whole-school approach to support effective communication between colleagues and with families, the teaching of social-emotional skills and strategies and planning for each student. Participants proposed that a whole-school approach to social-emotional learning enables them to provide students with consistency thereby improving the overall support students receive.

So, each week we have a professional learning meeting... and one of the agenda items is always student welfare. And so, we talk about how kids are going and if anyone else needs to be added and discussed. [school leader]

### Developing relationships with children

Several educators spoke about the need for a school culture that values staff developing relationships with children and encourages educators to be hands-on, accessible and tuned-in to children. Participants reported that a school culture that places importance on school leaders and classroom teachers knowing their students enhances their capacity to see when individual children are struggling and therefore better support them.

Because I spend a lot of time with the kids too, I know them well enough to know—it’s a quick, ‘Hi, how you going?’ and, ‘See you later, get back to class,’ or, ‘I can see you need some space. I’ll let you sit, and we’ll talk in a little bit about what’s going on’. [school leader]

### Provision of school-based resources

All educators spoke about the value of prioritising school-based services and resources to support children’s mental health and described a range of professionals and programmes that their schools had engaged to supplement what educators were able to do in classrooms. Participants’ schools had garnered a range of services and resources to support children’s mental health. Some had therapeutic services that came to the school site, others had implemented social-emotional learning programmes while others had on-site play, art or animal therapy for students. Some participant schools prioritised these strategies in their budget, others had engaged with support services that were eligible for subsidies from the government, and others had raised funds through community groups to subsidise their endeavours.

We do have an art therapist who comes in that works with some children. We pay for the ones that we believe are disadvantaged or the ones that the parents can’t afford, but there’s also a user-pay as well, so parents pay to work with him. [school leader]

### Impact on staff wellbeing

Another subtheme from several participants was how schools address the emotional impact experienced by educators working with children who have mental health difficulties. Participants spoke about the benefits of a school culture where educator wellbeing was prioritised, and that this improves their capacity to support children. Educators also spoke about the difficulties of working in a school where they receive very limited wellbeing support and that this compounds the pressures and difficulties of supporting students.

So, if there’s a child that’s disclosed something, it doesn’t just stop there. [The educators] carry that. [school leader]

...we’ve set up a really cool relaxation room... For the staff where they can just—if they need five minutes, they go and collect the key from the staff centre and they’ve got massage chairs and there’s just a bit of downtime, and then they get back into it... [school leader]

## External factors that influence school capacity

A theme among all participants was that a range of factors external to schools inhibits their capacity to support children’s mental health. Concerns included the inaccessibility of timely community-based and in-school child mental health support services and low child mental health literacy in the wider community.

### Inaccessibility of support services

All participants expressed frustration and concern about long waiting lists for assessment and intervention for children with mental health difficulties. Support services were consistently described as inaccessible; therefore, families were required to travel long distances to access help or were unable to afford private timely mental health services for their children in lieu of public services that had long waiting lists. Educators reported that a family’s inability to access professional mental health support is detrimental to the school’s capacity to effectively support children in the classroom. This is because they are left, with limited understanding, to care for children who may be experiencing significant mental health difficulties and exhibiting challenging internalising or externalising symptoms.

...even for them to get assessments done and things like that so that we can then help them so that we’ve got more knowledge. But 18 months on a waiting list sometimes it’s really hard... [classroom teacher]

...the waiting list is like a year plus. It’s a long time. And for us as a school, sometimes you can’t proceed with anything until you have a paediatrician. It’s not easy, you have to just—it’s a waiting game. [school leader]

### Insufficient support staff in schools

Another subtheme from most participants was the lack of access to allied health staff for assessment, intervention and support. Educators spoke of high allied health staff turnover, leading to frustration, inconsistency and miscommunication. Participants indicated that all primary schools should have ready access to support services, particularly to counsellors for children, but also health and allied health staff to assess and provide timely support and intervention.

Well, we lost our ed psych—they moved, and we were supposed to get a new one and they’ve just never eventuated. So, since the middle of the year, we probably haven’t had one allocated to us. So—which is interesting given that mental health is so prominent in the media and there’s so much about looking after children’s mental health. [classroom teacher]

### The need to improve child mental health literacy

Many participants reported that they thought child mental health literacy needs to be improved in the wider community. Educators reported that the stigma and lack of understanding about children’s mental health inhibit educators’ capacity to support children in the classroom because the lack of a common language around children’s mental health inhibits family engagement. Educators reported that when families are not engaged about their child’s mental health, it compromises what educators can do and means that some children may not get the support they need.

Just to bring the awareness ... So, the parents are not just saying, ‘Well, that’s just my child. He’s just grumpy all the time’. Well, because I mean, we see 20 of them and we can compare and we know the difference, whereas, that person might only have one child or two, so, and they only spend a couple of hours with them. [classroom teacher]

I think one of the biggest problems with kids with mental health issues is in primary schools. There’s still not really a huge recognition that kids can have [mental health issues]. [school leader]

## Discussion

This study indicates that despite being motivated to support children’s mental health, educators are feeling overwhelmed and pressured, out of their depth and frustrated by the complexities of their role. However, they have developed a range of informal strategies, using observation and relationship building, to identify and support children’s mental health needs. Educators provided insights into what they perceived as important for schools’ support of children’s mental health, such as school culture and partnering with families, while also indicating there are external influences that inhibit this support and expressing a need for a more effective approach to supporting children’s mental health.

While primary schools are increasingly viewed as a key community setting where children’s mental health can be supported, this study signals that educators have concerns about what this means for the complexities of their role, echoing research from secondary schools (Graham et al., 2011; Kidger et al., 2010; Mazzer & Rickwood, 2015; Shelemy et al., 2019). Indeed, the ‘complexities of the educator role‘ was a theme woven throughout all the focus groups and interviews. Similar to studies of educator perspectives in secondary schools, all participants thought it was important that schools address children’s mental health needs (Danby & Hamilton, 2016; Graham et al., 2011; Mazzer & Rickwood, 2015). However, educators felt overwhelmed by the needs of children’s mental health in their schools and classrooms (Walter et al., 2006). All participants had undertaken some child mental health professional development and were open to further training, but they felt they had too little time to do this because their teaching responsibilities meant they were time poor.

Educators consistently expressed concern about the pressures families are experiencing and the impact this has on children’s mental health, and on the school’s ability to engage and partner with families. While some participants had positive experiences of communication and collaboration with families, others had struggled because of the complexities of language, socio-cultural issues and low child mental health literacy among parents. This echoes other Australian studies about the concerningly low child mental health literacy levels among parents in the general community (Tully et al., 2019). This study indicates that child mental health literacy levels among parents not only affect children’s access to support services but importantly also inhibit the school’s capacity to provide support to children. Strategies to increase child mental health literacy in the community could support the identification of children’s mental health needs and the provision of appropriate support.

Participants commonly reflected on the importance of a school culture that prioritises mental health and takes a whole-school approach to social-emotional learning. However, participants spoke about a range of factors external to schools that inhibited their capacity to support the mental health needs of students. Concern about the long waiting lists for paediatric assessments wove through discussions of educator challenges about identifying and supporting children’s mental health needs. Participants spoke about many families who had waited over 12 months for an initial assessment of their child or faced prohibitively expensive private consultations. Participants expressed concern that when children do not get this help, educators, with limited capacity, are left to support these children in schools. This echoes the literature about the pressures on educators who grapple with being time poor and having limited mental health training (Shute & Slee, 2016). Educators felt that professional assessments of children’s mental health needs are invaluable to schools’ support of their students. However, they raised concerns that the school-based support services that exist for mental health needs are often inaccessible for children—either untimely or too expensive—and that this places significant pressures on educators to fill the gap. There was general agreement that school support services were often inaccessible or inadequate, characterised by frequent changes in allied health staff, who were limited in what they could provide.

Educators spoke about feeling uncertain about their skills for identifying children’s mental health difficulties (Graham et al., 2011; Kratt, 2017) stating that child mental health training would be useful, but that pressures in the classroom and school meant they often didn’t have time to do training or to implement training content. Rather, they saw improving child mental health literacy in the broad community, reducing waiting lists for professional children’s mental health assessments and providing better and more consistent access to allied health staff as vital to empowering schools to support children’s mental health.

This study raises implications for policies and practices within the wider education and health systems as well as in schools. Schools, and educators, require strategic support, by way of sufficient qualified support staff, if they are to effectively support children’s mental health. Addressing the mental health training needs of educators could build schools’ capacity to identify and support children’s mental health needs. However, this should not be done without addressing the time-poor nature of the primary school teacher role. Likewise, the health sector needs reform so that children and families can access mental health assessments and interventions in a timely manner, rather than waiting excessive amounts of time to get the help they need. When these concerns are addressed in parallel then the educator capacity to initiate care pathways for children can be supported so children can get the help they need.

## Strengths and limitations

The qualitative study design enabled exploration of the perspectives of primary school educators regarding supporting children’s mental health in schools. A question guide was used with flexibility, and open questions were asked to prompt depth of discussion. This enabled participants to express their views with latitude, not restricted to answering specific questions, and elicited a range of salient and poignant perspectives from participants that a more structured design might not have achieved (Kervin et al., 2006).

Because of the recruitment strategy where educators had to be proactive and contact the research team, only educators who were particularly interested in the topic participated. All participants were practising primary school educators who were interested in supporting children’s mental health and volunteered their time to the study. While the four participating schools varied geographically, they were all within a single region of the Department of Education and Training, Victoria. The results of this study were not intended to be generalisable to the wider population, and future research should be done in other areas to explore whether the themes generated from this study are shared by educators in other parts of Victoria.

This study was impacted when, because of the COVID-19 pandemic, the Department of Education and Training, Victoria suspended all research in schools within Melbourne for most of 2020. This meant that 12 schools, each with multiple participants, were withdrawn from the study. However, 17 participants is a suitable sample size for the study design and is comparable with other studies (Garvey et al., 2020; Shelemy et al., 2019).

## Suggestions for future research

Educators in this study spoke about the importance and complexities of engaging and partnering with families. Future exploration of families’ perspectives could contribute to clarifying and mitigating inhibitors to home–school partnerships and to elucidating how families see the role of schools regarding children’s mental health. Children’s voices are often absent in research that affects them and school mental health research is no exception (Fazel & Hoagwood, 2021). Research with child participants is important to give them a voice in matters that concern them, and so that school policies and practices can be sustained, informed and accepted by these pivotal stakeholders (Fazel & Hoagwood, 2021).

## Conclusion

While schools are increasingly viewed as an ideal setting to address children’s mental health needs (Patalay et al., 2017; Werner-Seidler et al., 2017), this study indicates that, despite being motivated, educators felt overwhelmed, pressured and out of their depth, and expressed a need for a more effective approach to supporting children’s mental health. This study elucidates primary school educator perspectives, including several suggestions to enhance primary schools’ capacity to support the mental health needs of children. These are as follows: that educators should receive pre-service, as well as ongoing, training in early identification of children’s mental health difficulties so that they can initiate care pathways for children; waiting lists for professional mental health assessments need to be addressed, so children and educators get appropriate and timely support; schools need access to regular, on-site, and ongoing support staff that can work with children and staff; and mental health literacy levels in the community need to be improved, to promote partnerships between schools and families by having a common language.

## Data Availability

N/A.

## Consolidated criteria for reporting qualitative studies (COREQ): 32 Item checklist (Tong et al., 2007)

**Table:**

No	Item	Guide questions/description	Reported on page #
	**Domain 1: Research team and reflexivity**		
	*Personal characteristics*		
1	Interviewer/facilitator	Which author/s conducted the interview or focus group?	p. 8
2	Credentials	What were the researcher’s credentials? E.g. PhD, MD	p. 8
3	Occupation	What was their occupation at the time of the study?	p. 8
4	Gender	Was the researcher male or female?	p. 8
5	Experience and training	What experience or training did the researcher have?	p. 8
	*Relationship with participants*		
6	Relationship established	Was a relationship established prior to study commencement?	p. 8
7	Participant knowledge of the interviewer	What did the participants know about the researcher? e.g. personal goals, reasons for doing the research	n/a
8	Interviewer characteristics	What characteristics were reported about the interviewer/facilitator? e.g. Bias, assumptions, reasons, and interests in the research topic	n/a
	**Domain 2: study design**		
	*Theoretical framework*		
9	Methodological orientation and Theory	What methodological orientation was stated to underpin the study? e.g. grounded theory, discourse analysis, ethnography, phenomenology, content analysis	p. 6
	*Participant selection*		
10	Sampling	How were participants selected? e.g. purposive, convenience, consecutive, snowball	pp. 6–7
11	Method of approach	How were participants approached? e.g. face-to-face, telephone, mail, email	pp. 6–7
12	Sample size	How many participants were in the study?	p. 7
13	Non-participation	How many people refused to participate or dropped out? Reasons?	p. 25
	*Setting*		
14	Setting of data collection	Where was the data collected? e.g. home, clinic, workplace	p. 8
15	Presence of non-participants	Was anyone else present besides the participants and researchers?	p. 8
16	Description of sample	What are the important characteristics of the sample? e.g. demographic data, date	pp. 6–7
	*Data collection*		
17	Interview guide	Were questions, prompts, guides provided by the authors? Was it pilot tested?	pp. 7–8
18	Repeat interviews	Were repeat interviews carried out? If yes, how many?	n/a
19	Audio/visual recording	Did the research use audio or visual recording to collect the data?	p. 8
20	Field notes	Were field notes made during and/or after the interview or focus group?	n/a
21	Duration	What was the duration of the interviews or focus group?	p. 8
22	Data saturation	Was data saturation discussed?	n/a
23	Transcripts returned	Were transcripts returned to participants for comment and/or correction?	p. 8
	**Domain 3: analysis and findings**		
	*Data analysis*		
24	Number of data coders	How many data coders coded the data?	p. 9
25	Description of the coding tree	Did authors provide a description of the coding tree?	pp. 9–10
26	Derivation of themes	Were themes identified in advance or derived from the data?	p. 9
27	Software	What software, if applicable, was used to manage the data?	p. 9
28	Participant checking	Did participants provide feedback on the findings?	p. 8
	*Reporting*		
29	Quotations presented	Were participant quotations presented to illustrate the themes/findings? Was each quotation identified? e.g. participant number	Yes pp. 10–20
30	Data and findings consistent	Was there consistency between the data presented and the findings?	Yes
31	Clarity of major themes	Were major themes clearly presented in the findings?	Yes pp. 10–20
32	Clarity of minor themes	Is there a description of diverse cases or discussion of minor themes?	Yes pp. 10–20

## Question guide for interviews and focus groups

**Table:**

When I say the term “child mental health” what does that mean to you?
Thinking about the school context, please tell me about what good mental health looks like in children?
What about children experiencing mental health concerns? Tell me about what that looks like, in a school context?
How do you identify if a child may need support for their mental health?
How do you determine what kinds of support children need, to develop good mental health?
How do you work with your students’ families to support the mental health of their children?
Thinking about the school context, in your experience, what do you think works well, in supporting children’s mental health?
Within the school context, what are the most common things you see getting in the way of children’s good mental health?
If you could change anything about the school system to better care for children’s mental health, what would you do?

## Code availability

N/A.
